# Asymptomatic progressive multifocal leukoencephalopathy: a case report and review of the literature

**DOI:** 10.1186/s13256-018-1727-7

**Published:** 2018-07-01

**Authors:** Yinan Zhang, Crystal Wright, Angela Flores

**Affiliations:** Department of Neurology and Neurotherapeutics, University of Texas Southwestern Medical Center, 5323 Harry Hines Blvd, Dallas, TX 75390 USA

**Keywords:** Multiple sclerosis, Natalizumab, Progressive multifocal leukoencephalopathy, Magnetic resonance imaging

## Abstract

**Background:**

We report the development of asymptomatic progressive multifocal leukoencephalopathy in a patient with multiple sclerosis on natalizumab therapy. Progressive multifocal leukoencephalopathy often presents with debilitating neurologic symptoms. Very few cases have documented a completely asymptomatic course of the disease.

**Case presentation:**

A 26-year-old white woman with multiple sclerosis was treated with natalizumab. She was diagnosed as having progressive multifocal leukoencephalopathy based on characteristic magnetic resonance imaging lesions after 27 infusions of natalizumab. She had no neurologic deficits at the time of diagnosis and John Cunningham virus in cerebrospinal fluid was detected at 15 copies/ml. She was initially treated with mefloquine and mirtazapine and remained asymptomatic for 3 months. She later developed worsening magnetic resonance imaging lesions related to immune reconstitution inflammatory syndrome. At that time, she received intravenously administered immunoglobulin and high-dose intravenously administered methylprednisolone with radiologic improvement of the lesions.

**Conclusions:**

Our case report illustrates that early detection of asymptomatic progressive multifocal leukoencephalopathy and its subsequent treatment resulted in a benign clinical course. In consideration of the additional small number of cases of asymptomatic progressive multifocal leukoencephalopathy that have been reported, we conclude that routine magnetic resonance imaging surveillance is important for patients with multiple sclerosis who are at high risk for developing natalizumab-associated progressive multifocal leukoencephalopathy.

## Background

Progressive multifocal leukoencephalopathy (PML) is the leading adverse effect from using natalizumab in the treatment of multiple sclerosis (MS). PML is a progressive multifocal disease involving the white matter and typically presents with subacute onset of symptoms including altered mental status, visual and motor deficits, and ataxia [1]. Natalizumab-associated PML carries an average mortality of 23% and survivors often develop debilitating neurologic deficits from the disease and its treatment sequelae, such as immune reconstitution inflammatory syndrome (IRIS), in which there is a paradoxical worsening of the infection due to overwhelming inflammatory reaction by the recovering immune system after discontinuing the immunosuppressing agent [2]. PML is caused by John Cunningham virus (JCV), and patients on natalizumab therapy receive serum JCV antibody index testing and surveillance with interval magnetic resonance imagings (MRIs) to assess PML risk and detect early stages of the disease. PML is seen on MRI as multifocal, asymmetric periventricular and subcortical lesions with minimal mass effect or enhancement [3]. Despite measures of clinical vigilance, PML often presents with new neurologic deficits prior to its diagnosis.

We report the case of a patient diagnosed as having PML based on characteristic MRI lesions from a routine surveillance scan who was clinically asymptomatic at the time of diagnosis and continued to have minimal disability throughout the course of the disease.

## Case presentation

**Fig. 1:**
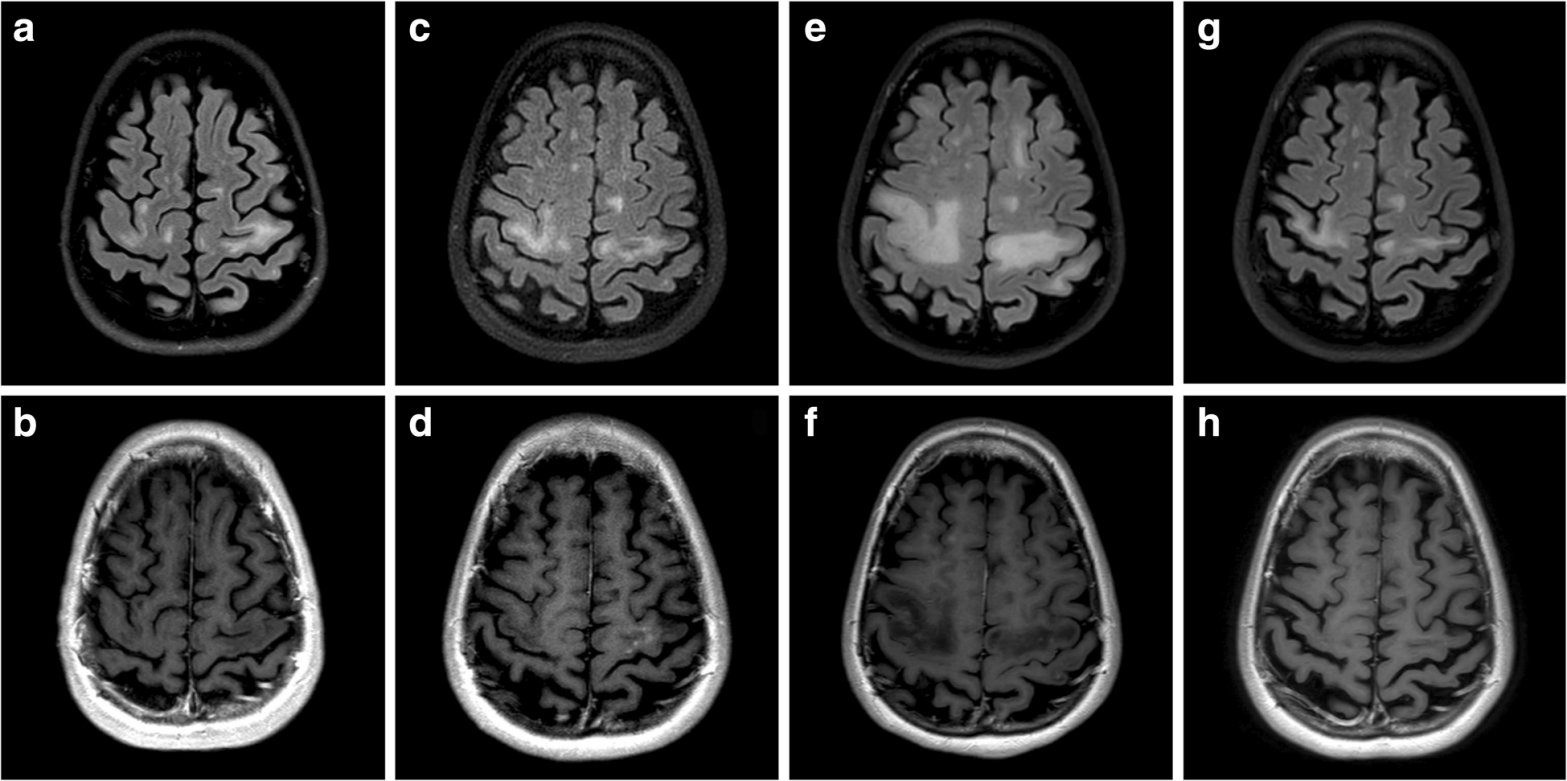
(**a**-**h**) Magnetic resonance imaging T2 fluid-attenuated inversion recovery and post-contrast T1 images of the patient at time of progressive multifocal leukoencephalopathy diagnosis showing bilateral asymmetric confluent non-enhancing hyperintensities in the subcortical precentral gyri (**a**, **b**), at 3 months showing a few small enhancing lesions in the left frontal lobe suggestive of immune reconstitution inflammatory syndrome (**c**, **d**), at 5 months showing interval development of T2 signal abnormality with mild enhancement (**e**, **f**), and at 1 year showing further decrease in T2 hyperintensities and resolution of enhancement (**g**, **h**)

A 26-year-old right-handed white woman with no significant medical history was diagnosed as having MS in 2013 at age 22 and experienced ongoing radiologic activity on both glatiramer acetate and dimethyl fumarate. She transitioned to natalizumab in July 2014 to stabilize disease activity, and her JCV antibody index was positive at 3.58 prior to starting natalizumab. She became clinically and radiologically stable with the initiation of natalizumab until November 2016 when a surveillance MRI of her brain showed asymmetric confluent non-enhancing hyperintensities in the bilateral subcortical precentral gyri consistent with PML (Fig. 1a, b). Cerebral spinal fluid (CSF) showed quantitative polymerase chain reaction (PCR) for JCV of 15 copies/ml, and other CSF studies were within normal limits. A diagnosis of PML was made based on the compatible neuroimaging findings along with the presence of JCV DNA in the CSF. Natalizumab was discontinued after 27 total treatments. Our patient was asymptomatic at the time of PML diagnosis, and she was highly functioning with an Expanded Disability Status Scale (EDSS) of 0. A decision was made to defer plasmapheresis at the time of diagnosis given her high functional status, subtle radiological change, and low viral titer. She was treated with orally administered mefloquine loading dose followed by 250 mg weekly and mirtazapine 15 mg daily.

Repeat MRI at 2 months following diagnosis showed no changes in her brain lesions. She remained asymptomatic until 3 months after diagnosis when she noticed mild dysmetria of her left hand that progressed to a tremor. The following month a repeat brain MRI revealed a few small enhancing lesions in her left frontal lobe suggestive of IRIS (Fig. 1c, d). The hyperintensities in the bilateral precentral gyri remained stable. Imaging of her cervical spine revealed a new non-enhancing cord lesion. She was then treated for 5 days with intravenously administered immunoglobulin and restarted on glatiramer acetate for MS treatment. A repeat CSF examination in February 2017 showed JCV PCR of 31 copies/ml.

Five months following her diagnosis, a repeat brain MRI showed interval development of T2 signal abnormality with mild enhancement in multiple areas including the brainstem, cerebellum, and bilateral cerebral hemispheres (Fig. 1e, f). A repeat lumbar puncture was performed. JCV PCR in the CSF was undetectable. Mefloquine and mirtazapine were discontinued. Given the MRI findings, she was treated for ongoing inflammation associated with IRIS versus a possible exacerbation of her underlying MS with high-dose intravenously administered methylprednisolone (IVMP) 1500 mg daily for 3 days. She was then transitioned from glatiramer acetate to ocrelizumab for treatment of MS. Six months following her diagnosis she reported changes in left hand dexterity and right upper extremity phasic spasms. A repeat lumbar puncture was performed and JCV PCR remained undetectable. She continued MRI surveillance followed by treatment with high-dose IVMP for a total of six courses until there was significant resolution of enhancement on her brain MRI (Fig. 1g, h). Following treatment, she has residual left hand dysmetria and tremor as well as right upper extremity phasic spasms. At 1-year follow-up, her EDSS is 2.0.

## Discussion

The patient described in this case report underwent an asymptomatic course of PML treated with mefloquine and mirtazapine. Due to the early discovery of PML in our patient and her intact neurologic examination at the time of diagnosis, we chose not to treat with plasma exchange (PLEX) in order to decrease the speed of immune reconstitution, which has been associated with stronger inflammation in patients with HIV-PML [4]. Further studies showed PLEX conferred no additional benefit of reduced mortality or improved outcome in patients who received PLEX (*n* = 184) versus those who did not (*n* = 35) for treatment of natalizumab-associated PML [5]. The neurologic deficits sustained toward the end of this patient’s treatment were thought to be from IRIS rather than the progression of PML lesions. While there is no definitive method of distinguishing PML lesions from those caused by IRIS [6], the presence of gadolinium enhancement on brain MRI along with undetectable JCV favored the diagnosis of IRIS rather than development of new PML lesions.

Although PML usually presents with clinical symptoms at the time of diagnosis, around 8% of patients with natalizumab-associated PML were asymptomatic at diagnosis, and PML lesions have been reported to present up to 6 months prior to onset of clinical symptoms [7, 8]. Almost all patients diagnosed as having PML develop neurologic deficits throughout the course of the disease with varying levels of severity. A retrospective case series of 336 patients with natalizumab-associated PML identified factors corresponding to improved survival and favorable outcomes including younger age and lower JCV count at diagnosis, low baseline disability at diagnosis, and localized MRI lesions [9]. Only a few cases have documented an asymptomatic disease course of PML (Table 1). While data from these reports are insufficient to conclude patterns leading to asymptomatic PML course, they illustrate the importance of MRI surveillance in detecting early radiologic evidence of PML prior to the development of clinical symptoms. The frequency of MRI monitoring has been debated and arguments against more frequent use cited the increased cost and limited imaging resources. However, reports have suggested 3–4 months as the optimal surveillance interval for patients at high risk of developing PML [10].

**Table 1:** Case reports of natalizumab-associated progressive multifocal leukoencephalopathy with asymptomatic disease course

Authors and Reference number	Age (years)/sex	EDSS at diagnosis	MRI findings	Development of IRIS	PML treatment	CSF JCV (copies/ml)	Risk factors [11]
This case report	26/F	0	Bilateral asymmetric confluent non-enhancing hyperintensities in the bilateral subcortical precentral gyri	Yes	Mefloquine and mirtazapine	15	27 natalizumab infusions, JCV-positive, JCV index 3.58
Blinkenberg *et al*. [12]	50/F	3.5	Right cerebellar peduncle hyperintense lesions	Yes	None, PML undiagnosed until presentation of PML-IRIS following natalizumab cessation	552	47 natalizumab infusions, JCV-positive
Fabis-Pedrini *et al.* [13]	24/F	6	Mildly enhancing small patchy lesions in left brainstem, cerebral peduncle, pontine tegmentum, and left brachium points	Yes	PLEX, mefloquine, mirtazapine, prednisone	63,910	Treatment with natalizumab for 54 months, JCV-positive
Mc Govern and Hennessay [14]	61/F	0	Left posterior parietal non-enhancing hyperintensity	No	PLEX, mirtazapine	12	Treatment with natalizumab for 3 years

*CSF* cerebrospinal fluid, *EDSS* Expanded Disability Status Scale, *F* female, *IRIS* immune reconstitution inflammatory syndrome, *JCV* John Cunningham virus, *MRI* magnetic resonance imaging, *PLEX* plasma exchange, *PML* progressive multifocal leukoencephalopathy

## Conclusions

When diagnosed early and treated, PML can present with a mild or even asymptomatic disease course. Clinical vigilance and routine MRI surveillance is important for patients with MS who are at high risk for developing natalizumab-associated PML.
